# Development of a PCL/gelatin/chitosan/β-TCP electrospun composite for guided bone regeneration

**DOI:** 10.1007/s40204-018-0098-x

**Published:** 2018-09-21

**Authors:** Masoumeh Ezati, Hamide Safavipour, Behzad Houshmand, Shahab Faghihi

**Affiliations:** 1 Stem Cell and Regenerative Medicine Group, National Institute of Genetic Engineering and Biotechnology (NIGEB), Tehran, 14965/161 Iran; 2 Department of Periodontics, School of Dentistry, Shahid Beheshti University of Medical Sciences, Tehran, Iran

**Keywords:** Guided bone regeneration, Electrospinning, β-Tricalcium phosphate (β-TCP), Composite membrane

## Abstract

Many approaches have been developed to regenerate biological substitutes for repairing damaged tissues. Guided bone/tissue regeneration (GBR/GTR) that employs a barrier membrane has received much attention in recent years. Regardless of substantial efforts for treatment of damaged tissue in recent years, an effective therapeutic strategy is still a challenge for tissue engineering researchers. The aim of the current study is to fabricate a GBR membrane consisting of polycaprolactone (PCL)/gelatin/chitosan which is modified with different percentages of β-tricalcium phosphate (β-TCP) for improved biocompatibility, mechanical properties, and antibacterial activity. The membranes are examined for their mechanical properties, surface roughness, hydrophilicity, biodegradability and biological response. The mechanical properties, wettability and roughness of the membranes are improved with increases in β-TCP content. An increase in the elastic modulus of the substrates is obtained as the amount of β-TCP increases to 5% (145–200 MPa). After 5 h, the number of attached cells is enhanced by 30%, 40% and 50% on membranes having 1%, 3% and 5% β-TCP, respectively. The cell growth on a membrane with 3% of β-TCP is also 50% and 20% higher than those without β-TCP and 5% β-TCP, respectively. Expression of type I collagen is increased with addition of β-TCP by 3%, while there is no difference in ALP activity. The results indicated that a composite having (3%) β-TCP has a potential application for guided bone tissue regeneration.

## Introduction

Serious traumas and infections may result in different types of defects in bone tissue. Several therapeutic and surgical approaches have been used for the repair and regeneration of alveolar bone such as distraction osteogenesis (Ilizarov 1989), bone grafts (Branemark 1983), osteoinduction (Reddi et al. 1987) and guided bone regeneration (GBR) (Chiapasco et al. 2009; Hämmerle and Karring 1998). Among these methods, GBR, a surgical procedure that employs a barrier membrane, has the most predictable results for regeneration of bone tissue into the defect site. The membrane acts as a physical barrier to create an excluded space around the defect by preventing the invasion of fibrous connective and epithelial tissues into the defect which allows bone tissue to regenerate naturally (Dahlin et al. 1989; Xu et al. 2012). Common materials that are used for fabrication of barrier membranes can be divided into non-absorbable polytetrafluoroethylene (e-PTFE) (Lindfors et al. 2010; Lu et al. 2012; Urban et al. 2009), titanium mesh (Degidi et al. 2003) and bio-absorbable (collagen, chitosan (Coïc et al. 2009; Jiang et al. 2015), poly(glycolic acid) (PGA), poly(lactic acid) (PLA), poly(*ε*-caprolactone) (PCL) (Schmidmaier et al. 2006) inorganic ceramics (Kinoshita et al. 2008). For a successful bone regeneration, barrier membranes should possess some fundamental requirements including biocompatibility, biodegradability, sufficient mechanical strength, space maintenance, manageability and adhesiveness toward surrounding bone tissues (Gottlow 1993; Smith et al. 2009). To have a membrane that meets these requirements, a combination of two or more polymers should be used.

Many researchers have investigated GBR membranes which are fabricated from a combination of different polymers. Ke Ren et al. (2017) reported an improved cell adhesion and proliferation on nanofibrous PCL/gelatin GBR membrane made by the electrospinning process due to an increased wettability. Sarasam et al. (2005) used PCL/chitosan membrane that improved the mechanical properties and cell viability compared to pure chitosan. Chen et al. made biodegradable poly(L-lactide) (PLLA)/chitosan membranes for guided periodontal tissue regeneration and found that modification of chitosan could promote hydrophilicity, enhance cell proliferation and accelerate the degradation rate of PLLA electrospun membranes (Cao et al. 2013). A β-tricalcium phosphate (β-TCP)–collagen composite membrane has been reported to hold greater osteoconductivity and better biodegradation properties than commercial Bio-Oss collagen membrane (Kato et al. 2014). Lam et al. investigated the degradability of a PCL/β-TCP scaffold in comparison to pure PCL which revealed that addition of β-TCP could decrease the surface wettability which in turn resulted in increased degradability of the scaffold (Heidemann et al. 2001).

These studies clearly show that by taking advantage of two or more polymers, higher osteoconductivity, biodegradability and better mechanical properties could be achieved. Gelatin has several superior properties such as good biocompatibility, low immunogenicity, good tissue integration and hemostatic property, which makes it a great material for GBR membranes (Jiang et al. 2015; Postlethwaite et al. 1978). Chitosan, as a suitable natural polymer candidate, possesses many advantages such as low cost, biocompatibility, biodegradability, weak immunogenicity and most importantly antimicrobial characteristics (Kong et al. 2010; Mota et al. 2012; Xu et al. 2012). However, chitosan exhibits low mechanical strength (Hürzeler et al. 1998; Ko et al. 2010) and a high degradation rate (Kong et al. 2010). PCL has been also extensively investigated in bone tissue engineering due to its good biocompatibility, biodegradability and good mechanical properties, but displays low wettability (Bosworth and Downes 2010), poor cell adhesion (Kim et al. 2006; Li et al. 2006) and slow biodegradation rate (Jeong et al. 2004; Li et al. 2006).

Although chitosan/PCL, gelatin/PCL and PCL/TCP membranes have been studied previously, there is no report on PCL/gelatin/chitosan membrane that particularly formed composites with β-TCP in GBR applications. In this study, composite substrates by combining poly(*ε*-caprolactone), gelatin, chitosan and different ratios of β-TCP (0, 1%, 3% and 5% w/v) were fabricated by the electrospining method. β-TCP is added to the substrates as it has demonstrated good osteoinductivity, excellent biocompatibility and bioactivity (Zhou and Lee 2011). β-TCP can also serve as a precursor for Ca^2+^ and PO4^3−^ ions that encourage new bone formation (Lam et al. 2007). The characteristics of fabricated substrates including mechanical properties, elemental makeup, surface roughness and wettability are examined. In vitro cell culture experiments by human osteoblast-like cells MG63 are performed to evaluate cell adhesion, proliferation, alkaline phosphatase activity and collagen I gene expression on the surface of composite samples. Also, in vitro degradation behavior, antibacterial activity and bacterial adhesion of samples are investigated.

## Materials and methods

### Materials

Chitosan (medium molecular weight), polycaprolactone (PCL) (M_n_ 80,000 g/mol), gelatin and β-TCP (particle size < 0.063 µm) were purchased from Sigma-Aldrich. Dulbecco’s Modified Eagle Medium (DMEM). MTT [3-4,5-dimethylthiazol-2yl(-2,5diphenyl-2H-tetrazoliumbromide)], fetal bovine serum (FBS), acetic acid and formic acid were purchased from Sigma-Aldrich.

### Composites formation

The PCL (5% w/v) solution was prepared in acetic acid/formic acid (1:1, v/v) solvent mixture by stirring the mixture at 500 rpm for 2 h, and gelatin (5% w/v) was dissolved in acetic acid/formic acid (1:1, v/v) by stirring the mixture at 500 rpm for 3 h. Similarly, chitosan solution (3% w/v) was prepared in acetic acid at 500 rpm for 2 h. All the polymeric solutions were prepared at room temperature (37 ± 1 °C). After preparation of polymeric solutions, PCL (40 wt%), gelatin (40 wt%) and chitosan (20 wt%) were mixed to obtain a stock solution. Different ratios of β-TCP (0, 1%, 3% and 5% w/v) were added to the final uniform polymeric solution under slow magnetic stirring for 24 h. After 24 h, an immiscible polymeric blend of PCL/gelatin/chitosan/β-TCP was obtained which was used for electrospinning.

Nanofibrous substrates from PCL/chitosan/gelatin/β-TCP (0, 1%, 3% and 5%) were fabricated using electrospinning (ES). The polymer blend of PCL, chitosan, gelatin and β-TCP (0, 1%, 3% and 5%) was loaded into a 5 mL plastic syringe with a 22G×  32 mm needle and injected using a syringe pump. The flow rate of the polymer solution was maintained at 0.9 mL/h. A high voltage of 10 kV was applied at the tip of the needle and a distance of 10 cm between needle and collector was sustained throughout the process. The electrospinning process was carried out at 25 ± 1 °C and humidity of 45%. Substrates were collected on a flat aluminum plate and cross-linked with EDC and *N*-hydroxyl succinimide (NHS). Next, 2.5 mM EDC and 1.25 mM NHS were dissolved in ethanol/PBS solution (80/20) and the substrates were placed in the prepared solution for 1 h. Cross-linked substrates were washed thoroughly with deionized water to remove excess EDC/NHS. All the samples were gamma-sterilized for 2 h prior to biological experiments and referred as PGC (PCL/chitosan/gelatin), PGCT_1_ (PCL/chitosan/gelatin plus 1% β-TCP), PGCT_3_ (PCL/chitosan/gelatin plus 3% β-TCP) and PGCT_5_ (PCL/chitosan/gelatin plus 5% β-TCP).

### Composite sample characterization

After fabrication of composite samples, surface wettability, surface roughness, mechanical properties, chemical composition and morphology were characterized. The surface wettability of PGC, PGCT_1_, PGCT_3_ and PGCT_5_ was determined by measuring the contact angles of deionized water on each sample using a surface analysis system equipped with Image Analyzer software (OCA 15 plus; Data physics(. An autopipette was used to ensure a uniform volume of water droplets (0.5 µL). The experiments were run at room temperature on four membrane samples at three different times. The surface roughness of the samples was evaluated through AFM) Auto; Probe; Veeco (in tapping mode. The mechanical properties of the substrates were analyzed with a universal materials machine at room temperature with a cross-head speed of 5 mm/min. Rectangular samples with dimensions of 3 × 0/5 cm^2^ were prepared (*n *= 5) and used for the measurements. The elemental composition of each sample’s surface was evaluated using an energy-dispersive X-ray spectrometer (EDAX) to explore calcium and phosphate presence and distribution of particles. Chemical analysis of all components was performed by Thermo Nicolet FTIR (Nexus, USA) spectroscopy over a range of 4000 and 500 cm^−1^. The morphology of PGC, PGCT_1_, PGCT_3_ and PGCT_5_ membrane samples was examined using (EM3200/KYKY) scanning electron microscopy (SEM). Before imaging, samples were coated with gold for 30 min using a sputter coater for 60 s at an accelerating voltage of 12 kV. Fiber diameter of samples was studied based on SEM images at 5000× magnification. Five images were used for each sample and 40 different fibers were randomly selected. The average fiber diameter was calculated using Image Analysis software (Image J, NIH, USA). The pore size of fabricated samples was also analyzed based on SEM images.

### Cell culture

MG63 osteoblast-like cell line was used for this study and cultured in DMEM supplemented with 10% fetal bovine serum (FBS) and 1% penicillin/streptomycin at 37 °C in a 5% CO_2_. The culture medium was changed every 2 days. For biological experiments, cultured cells were detached by trypsinization, suspended in new culture medium and used for designed experiments.

### Cell attachment and proliferation

MTT assays determine the ability of mitochondrial dehydrogenases enzymes of living cells to oxidize a tetrazolium salt [3-(4,5-dimethylthiazolyl-2-y)-2,5 diphenyltetrazolium bromide] into an insoluble purple formazan product. The concentration of the purple formazan product was directly proportional to the number of metabolically active cells. MTT assay was performed to study the attachment and proliferation of MG63 cells on PGC, PGCT_1_, PGCT_3_ and PGCT_5_ samples. The sterilized samples were placed in a 48-well culture plate and seeded with 50,000 cell/mL for adhesion and 15,000 cell/mL for proliferation and incubated for different time points for each test (2, 4 and 6 h for adhesion, 1, 3 and 7 days for proliferation). Cells cultured in culture plates were used as control. After each time point, the samples were transferred into a new culture plate and washed with phosphate-buffered saline (PBS) to remove the unattached cells. Freshly prepared media of complete DMEM and 10 µl of MTT solution (5 mg/mL stock in 1 × fresh medium) were added into each well to make a final volume of 100 µL. The plate was placed in a CO_2_ incubator for 3 h until purple color of formazan crystals was formed. After 3 h, the formazan crystals were dissolved in solubilizing solution and transferred into a 96-well plate. The absorbance was measured at a wavelength of 570 nm with subtraction of 650 nm background using a UV–Vis spectrophotometer. A standard curve was drawn to estimate the cell number.

### Cell morphology

Cell morphology of MG63 on PGC, PGCT_1_, PGCT_3_ and PGCT_5_ substrates was studied by SEM analysis. Sterilized membrane samples were placed in 48-well culture plates and seeded with 4 × 10^3^ cells and incubated for 2 days. Following the incubation period, samples were rinsed three times with PBS and fixed with 2.5% glutaraldehyde, dehydrated with gradient concentration of ethanol (30, 40, 50, 60, 70, 80, 90 and 100%). Finally, they were air-dried overnight sputter-coated with gold and examined with scanning electron microscopy (SEM; EM3200) for investigating cell morphology.

### DNA content of grown cells

DNA quantification was performed to study the cell growth on PGC, PGCT_1_, PGCT_3_ and PGCT_5_ substrates. The samples were placed in 48-well culture plate, seeded with 15 × 10^3^ cells and incubated for 1, 3 and 5 days. After the incubation period, samples were rinsed with PBS and DNA content of cells was isolated with lysis buffer (10%Triton X-100, 5% Tween 20, 100 m mol^−1^ Tris–Hcl (pH 8), 10 mmol^−1^ EDTA). A NanoDrop 1000 spectrophotometer (Thermofisher, USA) was used to calculate the total DNA content of cells based on the read at absorbance wavelength of 260 nm.

### Gene expression

Reverse transcription polymerase chain reaction (RT-PCR) was used to analyze the expression level of type I collagen and alkaline phosphatase on the surface of the samples. The cells were seeded on the samples in a 24-well culture plates (control group) at an initial density of 10,000 cells/mL and cultured for 14 and 21 days. The cells were then lysed with TRIzol reagent (Invitrogen). The total RNA concentration and purity were determined from the value of the absorbance at 260 and 280 nm using a Nanodrop spectrophotometer. The extracted RNA was then stored at − 70 °C. The primers that are designed and used for this experiment are shown in Table 1. Complementary DNA (cDNA) was synthesized using 1 μg of total RNA primed with oligo (dT).

**Table 1:** Roughness and contact angle measurement of samples

Sample	Angle (°)	Ra (nm)
PGC	34.55 ± 0.56	52.55 ± 1.2
PGCT_1_	32.42 ± 0.11	61.12 ± 1.8
PGCT_3_	26.78 ± 0.34	69 ± 1.3
PGCT_5_	25.54 ± 0.48*	79.24 ± 1.4**

*P < 0.05 compared to other samples, **P < 0.05 compared to other samples

### Antibacterial activity of the membranes

In this study, the antibacterial activity of the PGC, PGCT_1_, PGCT_3_ and PGCT_5_ membrane samples was evaluated by agar disc diffusion method against *E. coli* and *S. aureus*. For disc diffusion, bacteria suspension with concentration in the range of 1.5 × 10^8^ UFC/mL was seeded by swabbing evenly in three directions to form a lawn. Sterile samples with 9 mm diameter along with control disc were placed on the surface of each inoculated MHA (Mueller–Hinton agar). The plates were incubated at 37 °C for 24 h and inhibition zones were observed.

Microbial adhesion (accumulation) and connectivity on a substrate can be observed by scanning electron microscopy (SEM). For this purpose, substrates were exposed to bacterial suspension for 6 and 72 h. After each time point, the samples were washed with PBS, fixed with 2.5% glutaraldehyde for 24 h, dehydrated in graded ethanol series (30, 40, 50, 60, 70, 80, 90 and 100), dried in a vacuum oven overnight, sputter-coated with gold and examined with scanning electron microscopy (SEM; EM3200).

### In vitro degradation and swelling

In vitro degradation assay was performed by incubating the samples in 4 mL PBS solution at pH of 7.4 and 37 °C up to 4 weeks. At weekly intervals, the samples were removed from the PBS solution and rinsed with distilled water and dried in a vacuum oven at 25 ^°^C. The weight retention was calculated using Eq. (1).

1 $${\text{Degradation}}\;(\% ) = \frac{{W_{0} - W_{t} }}{{W_{0} }} \times 100,$$

where the sample’s weight before soaking in PBS is (*W*_0_) and the sample’s weight after specific soaking time is (*W*_*t*_).

Changes in the sample weights and pH during in vitro degradation tests were measured. After 4 weeks, samples were removed from the solution and morphological evaluation of the samples was done with SEM.

The extent of swelling can be a measure of nutrient exchange by substrates. Samples with precisely measured size and weight were immersed in phosphate-buffered saline (PBS) for 5 h. After each 15 min, the samples were removed from the solution and placed on a filter paper to remove the excess water. The sample weights were then precisely measured. Swelling percent of samples was calculated according to Eq. (2).

2 $${\text{Swelling}}\;(\% ) = \frac{{W_{s} - W_{d} }}{{W_{d} }} \times 100,$$

where *W*_d_ is the dry weight and *W*s is the weight of the samples after swelling.

### Statistical analysis

All data are presented as the mean ± SD of at least three experiments. Statistical analysis was performed with GraphPad Prism software (GraphPad, San Diego, CA, USA) using a two-way ANOVA followed by Tukey’s multiple comparison test. The results were considered statistically significant when *p *< 0.05.

## Results and discussion

### Composites characterization

**Fig. 1:**
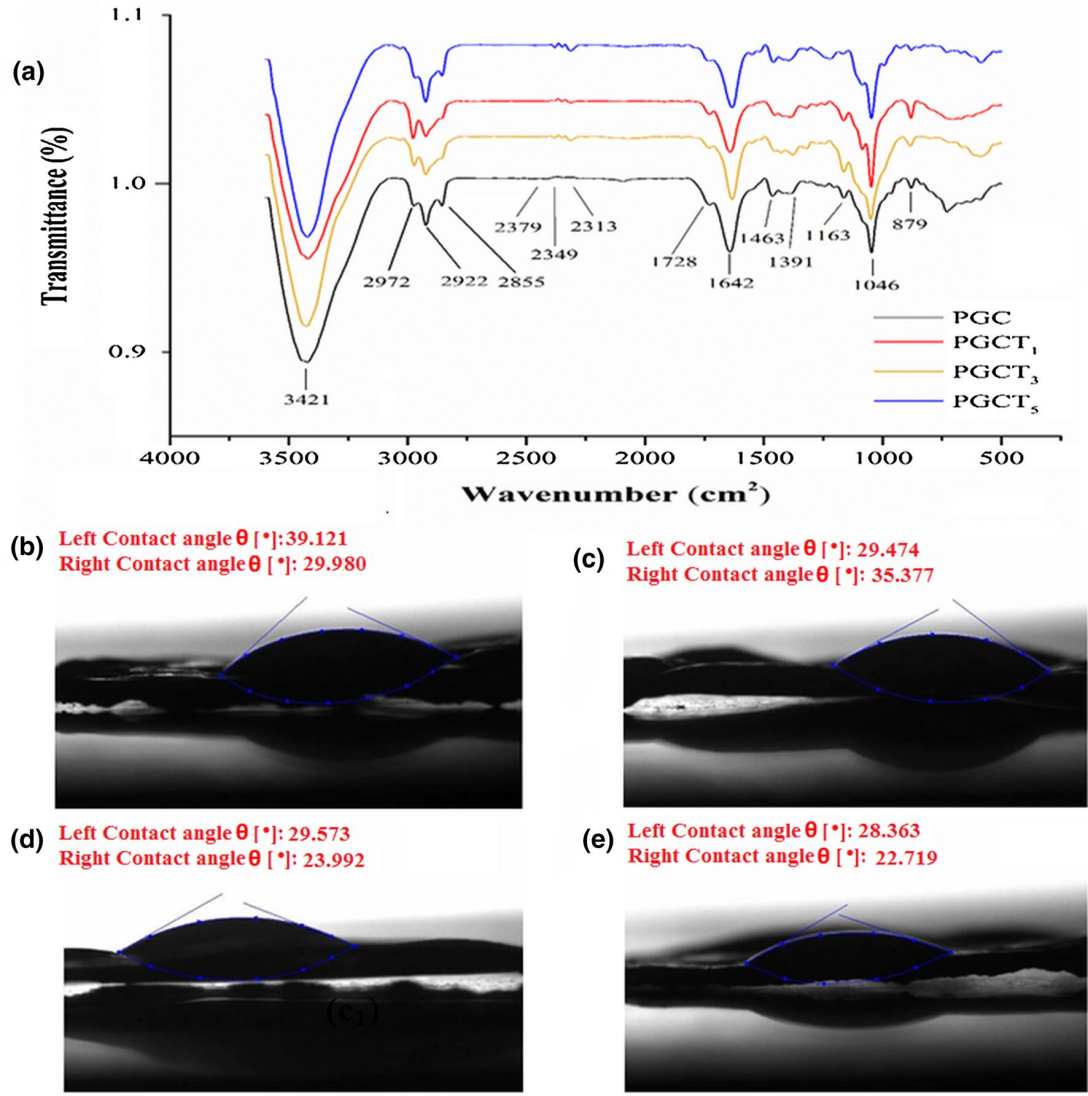
FTIR spectra of the samples (**a**). Water contact angle measurements of PGC (**b**), PGCT_1_ (**c**), PGCT_3_ and (**d**) PGCT_5_ (**e**) samples

The FTIR spectra of substrates containing different percentages of β-TCP nanoparticles confirm the presence of all three polymers in the fabricated membranes Fig. 1. The peaks of 2922, 2865 and 1728 cm^−1^ are correlated to PCL which correspond to asymmetric CH_2_, symmetric CH_2_, carbonyl group and asymmetric COC, respectively. The characteristic peaks at 1463, 1391 and 1046 cm^−1^ correspond to amidi I, amidi III and COO (backbone vibration) of chitosan, respectively. Gelatin presence was confirmed by a strong peak at 1642 cm^−1^ related to amidi I bond. β-TCP particles were confirmed by the presence of strong peak at 1163 cm^−1^ representing the distributed strong bond in the phosphate group (PO_4_^3−^).

The water contact angles of the substrates are presented in Table 1. The results clearly show that surface wettability was significantly increased with increase in β-TCP concentration (Fig. 1). The contact angle of the samples is considered one of the physical parameters which could relate the affinity of cells and proteins onto a surface.

**Fig. 2:**
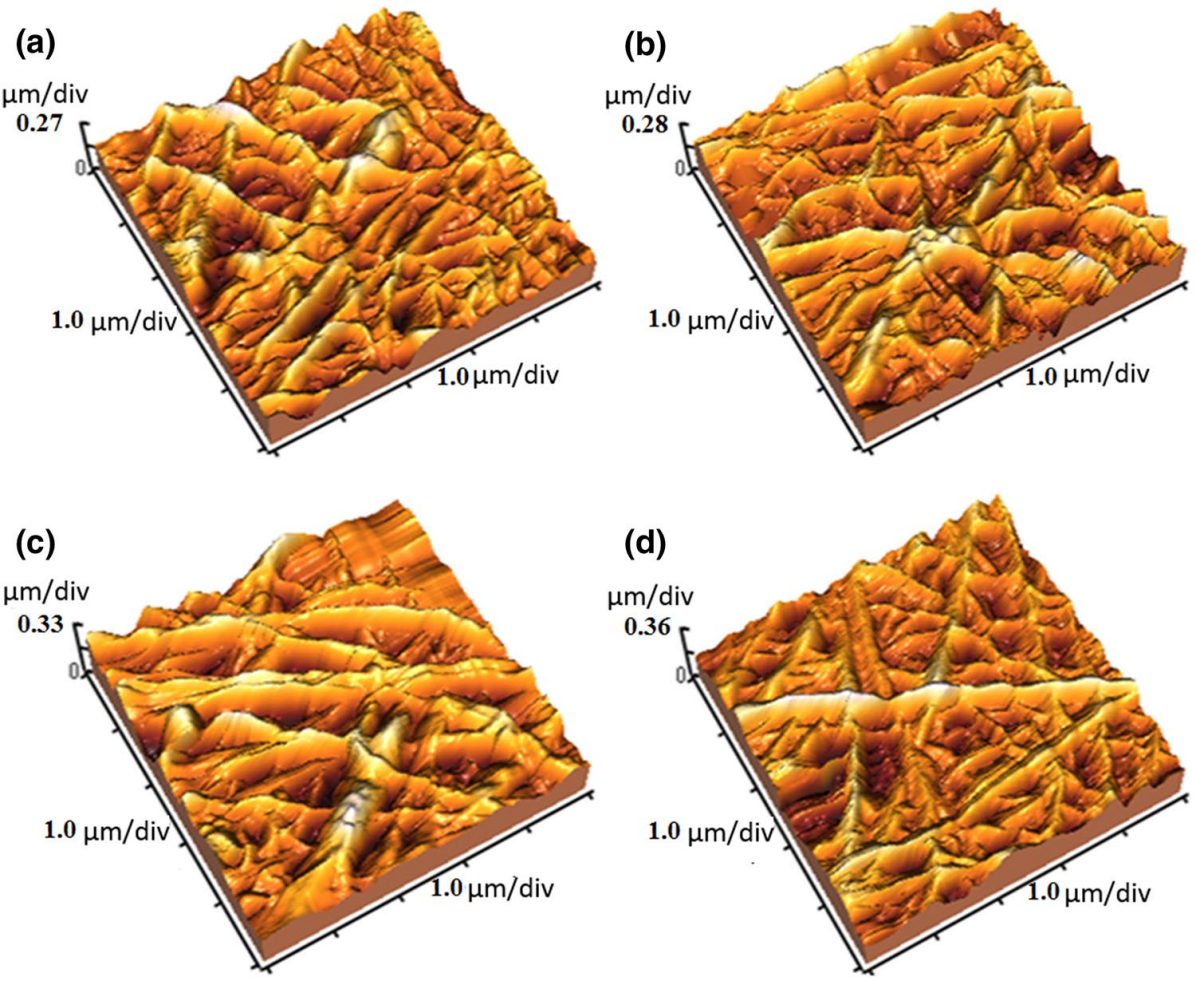
AFM images of PGC (**a**), PGCT_1_ (**b**), PGCT_3_ (**c**) and PGCT_5_ (**d**) samples

The surface roughness and topography of substrates were evaluated by AFM. The images and the surface roughness values are shown in Fig. 2 The results show that the addition of β-TCP into the modified PGC resulted in the enhancement of surface roughness (Table 1).

### SEM analysis

**Fig. 3:**
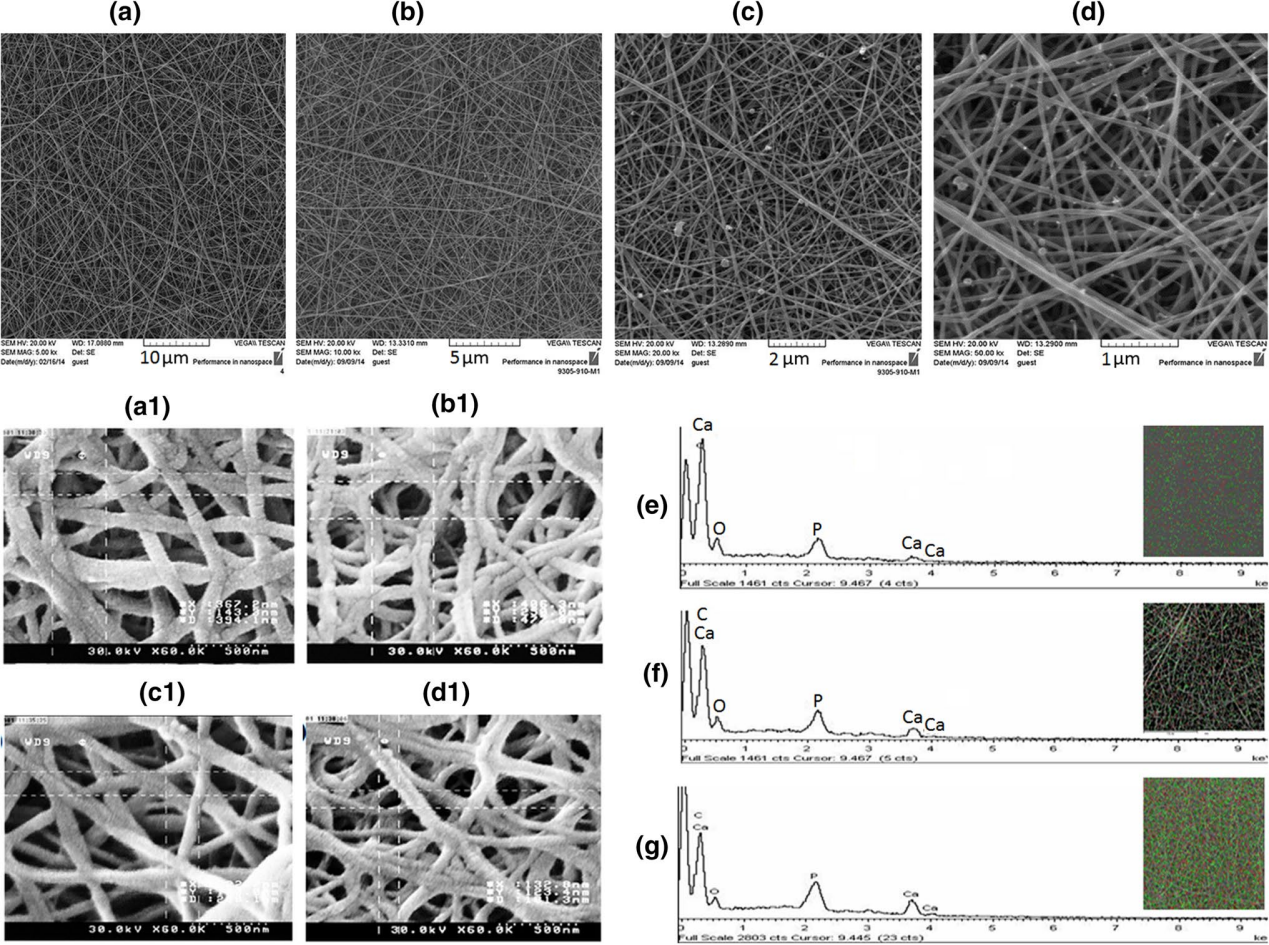
FE-SEM micrographs of PGC (**a**), PGCT_1_ (**b**), PGCT_3_ (**c**) and PGCT_5_ (**d**) samples, pore size of PGC (**a**_**1**_), PGCT_1_ (**b**_**1**_), PGCT_3_ (**c**_**1**_) and PGCT_5_ (**d**_**1**_). The EDAX pattern of PGCT_1_ (**e**), PGCT_3_ (**f**) and PGCT_5_ (**g**). The insets show the distribution of Ca and P ions on the surface of samples

Figure 3a–d shows the SEM images of composite substrates’ morphology indicating smooth and bead-free fibers. It is evident that voltage, distance between the needle and collector as well as the flow rate of polymer solution have been optimized. The images also clearly show the presence of tricalcium phosphate on the surface of substrates. EDAX analysis was also carried out to confirm the extent of P and Ca elements and the distribution of β-TCP particles Fig. 3e. The results showed that increase in the percentage of TCP led to the extension of P and Ca in the substrates. The results also confirmed a homogenous distribution of particles on the substrate surfaces. As the fiber diameter has been shown to be an important parameter for the growth of osteoblast cells over the scaffold (Takahashi and Tabata 2004), the fiber diameter and pore size of substrates were studied using SEM. The fiber diameters of the samples spanned 200–500 nm with an average fiber diameter of 291 nm. Pore size is another key factor for cell migration into the scaffold which can facilitate efficient exchange of nutrients and metabolic function between the scaffold and its environment (Akin et al. 2001; Takahashi and Tabata 2004). Differences in the extent of bone regeneration have been observed depending on the pore size of scaffolds (Pineda et al. 1996). It was observed that the average pore size was reduced by increasing the percentage of β-TCP. According to the results, PGC and PGCT_5_ revealed the highest and the lowest pores, respectively Fig. 3a_1_–d_1_.

### Mechanical characterization

**Fig. 4:**
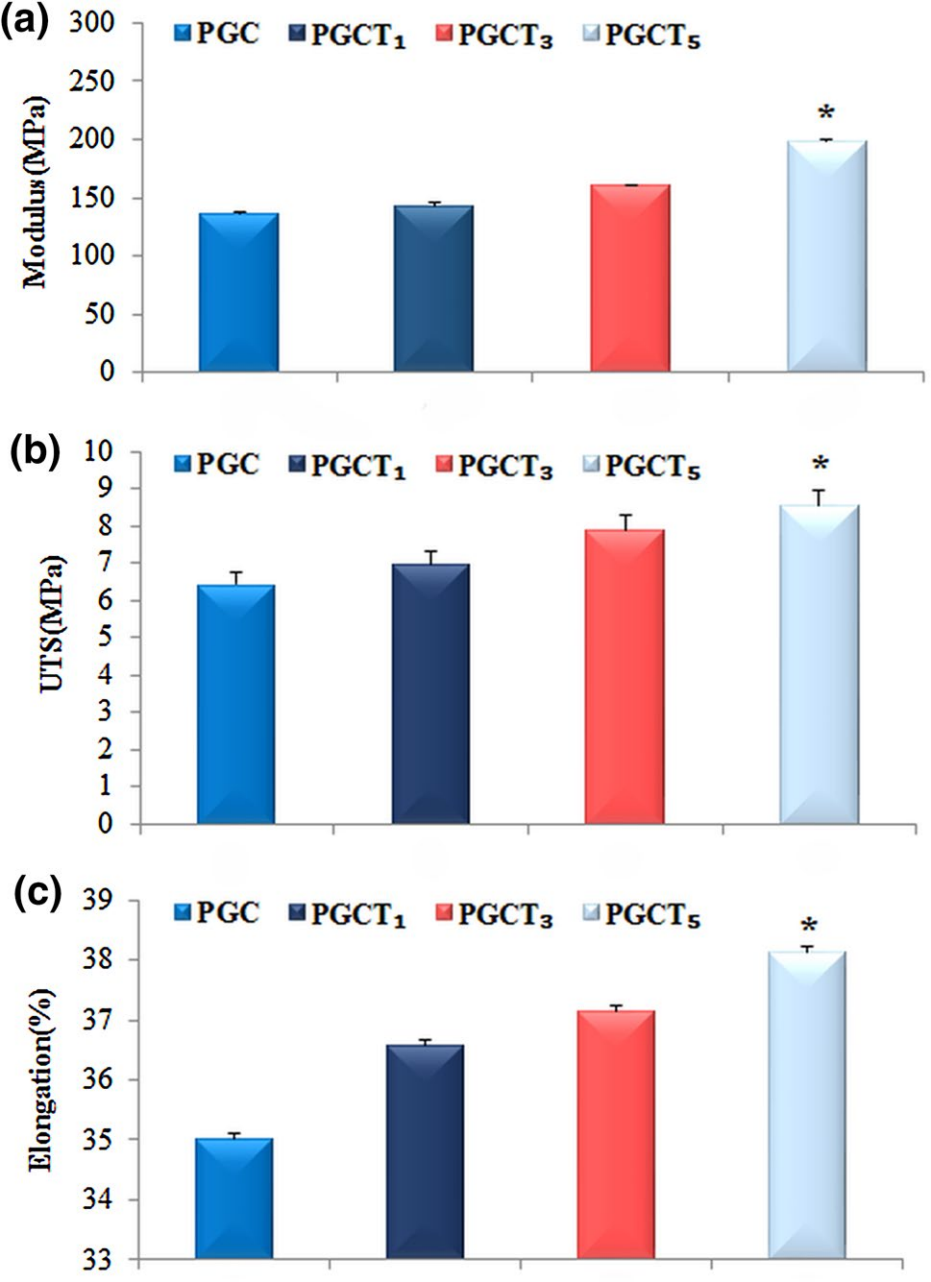
Mechanical properties of samples in wet condition including elastic modulus (**a**), ultimate tensile strength (**b**) and elongation (**c**). *P < 0.05 compared to the other samples

Having good mechanical properties is an essential requirement for a barrier substrate which would be an asset during bone formation (Lu et al. 2012). The tensile mechanical properties of substrates were investigated by their respective stress–strain curves. The values of Young’s modulus and compressive strength of the nanocomposite samples are illustrated in Fig. 4. As the amount of β-TCP increased up to 5%, the elastic modulus, elongation and ultimate tensile strength of substrates were significantly enhanced. The improvement of mechanical properties can be attributed to increase of β-TCP, since previous works have shown that β-TCP can have a significant positive effect on the mechanical properties (Tsuru et al. 2013). This effect comes from the creation of cross-linking between polymer chains which leads to a decrease of overall pore size (Aryaei et al. 2015; Sheikh et al. 2015; Maji and Dasgupta 2017). However, an increase in pore size may result in weaker mechanical properties (Dimitriou et al. 2012).

### Cell attachment and proliferation

**Fig. 5:**
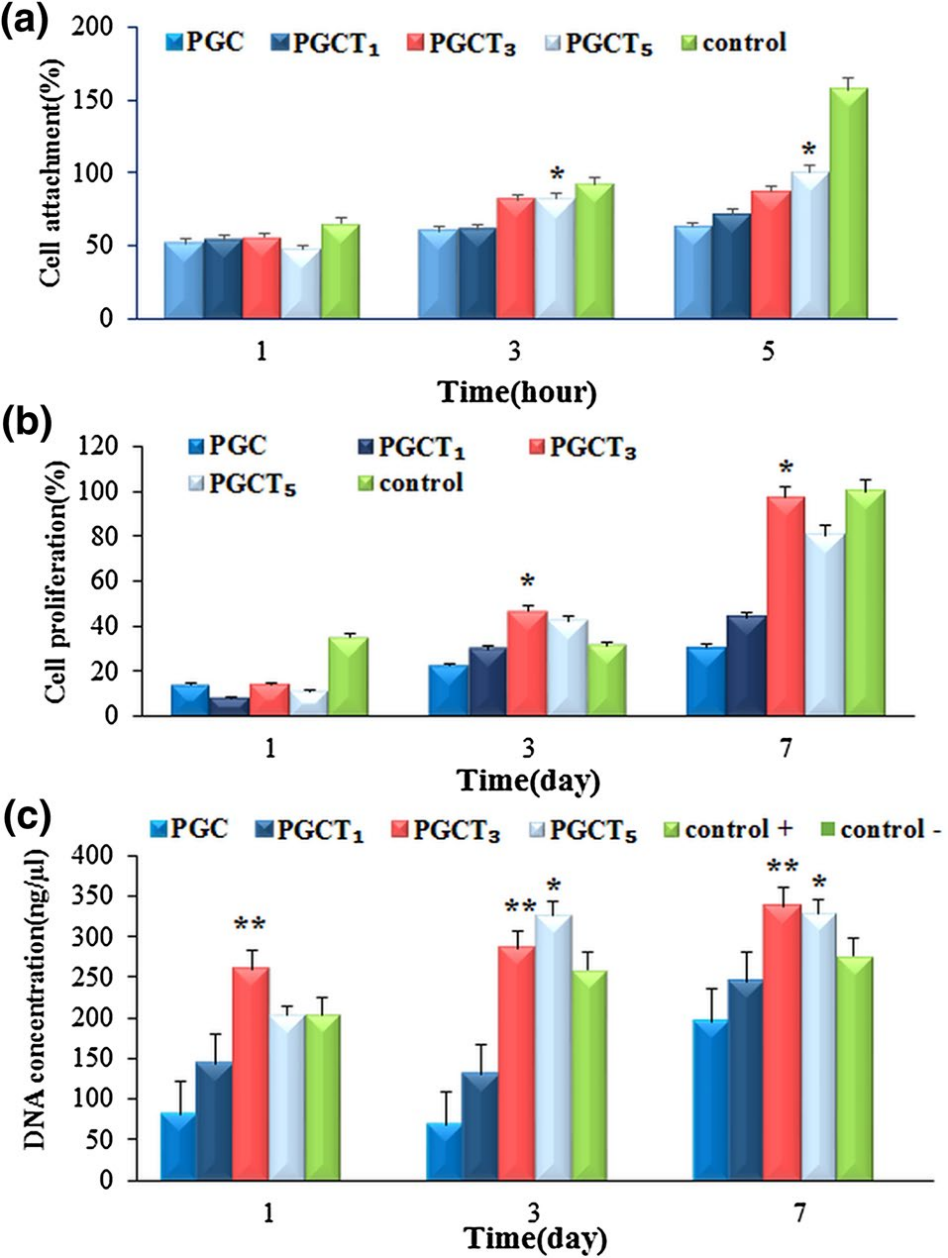
The attachment of MG63 cells on the composites containing different percentages of TCP (**a**); *P < 0.05 compared to PGC, PGCT_1_ and PGCT_3_. The histograms representing cell proliferations on different samples (**b**); *P < 0.05 compared to PGC, PGCT_1_ and PGCT_5_. DNA concentration of adhered cells on the samples (**c**); *P < 0.05 compared to PGC, PGCT_1_ and **P < 0.05 compared to PGC, PGCT_1_

MTT assay includes a reduction reaction in which reduced MTT reagent emerges with a blue formazan when incubated with viable cells and the formazan absorption indirectly reflects the level of cell viability. Many studies have shown that β-TCP has good biocompatibility and osteoconductivity (Nandakumar et al. 2009; Shim et al. 2012). The biological role of β-TCP particles on PGC, PGCT_1_, PGCT_3_ and PGCT_5_ samples was assessed in terms of cell adhesion and proliferation. The results of MTT assay demonstrated that the affinity of cells for PGC was inferior compared to cell affinity toward PGCT_1_, PGCT_3_ and PGCT_5_ membrane samples owing to the latter’s higher hydrophilicity and roughness. Cell adhesion increased on PGCT_1_, PGCT_3_ and PGCT_5_ after 5 h, but the increase in cells on PGCT_5_ was significantly higher than the others. As indicated in Fig. 5a, the culture plate was used as control.

Cell proliferation on the membrane samples was also evaluated through MTT assay and DNA content measurement after cultivation of cells for 1, 3 and 7 days (Fig. 5b). After 1 day of culture, the number of cells on PGC was lower compared to other samples. After 3 days, PGCT_3_ and PGCT_5_ showed slightly higher cell number and DNA concentration than PGC and PGCT_1_ membranes. After 7 days of culture, the cell number on PGCT_3_ was significantly higher than that of PGCT_5_. These results suggested that 3% w/v of β-TCP could have a positive effect on the proliferation rate of the cells in longer periods of culture) Fig. 5c).

### Cell morphology

**Fig. 6:**
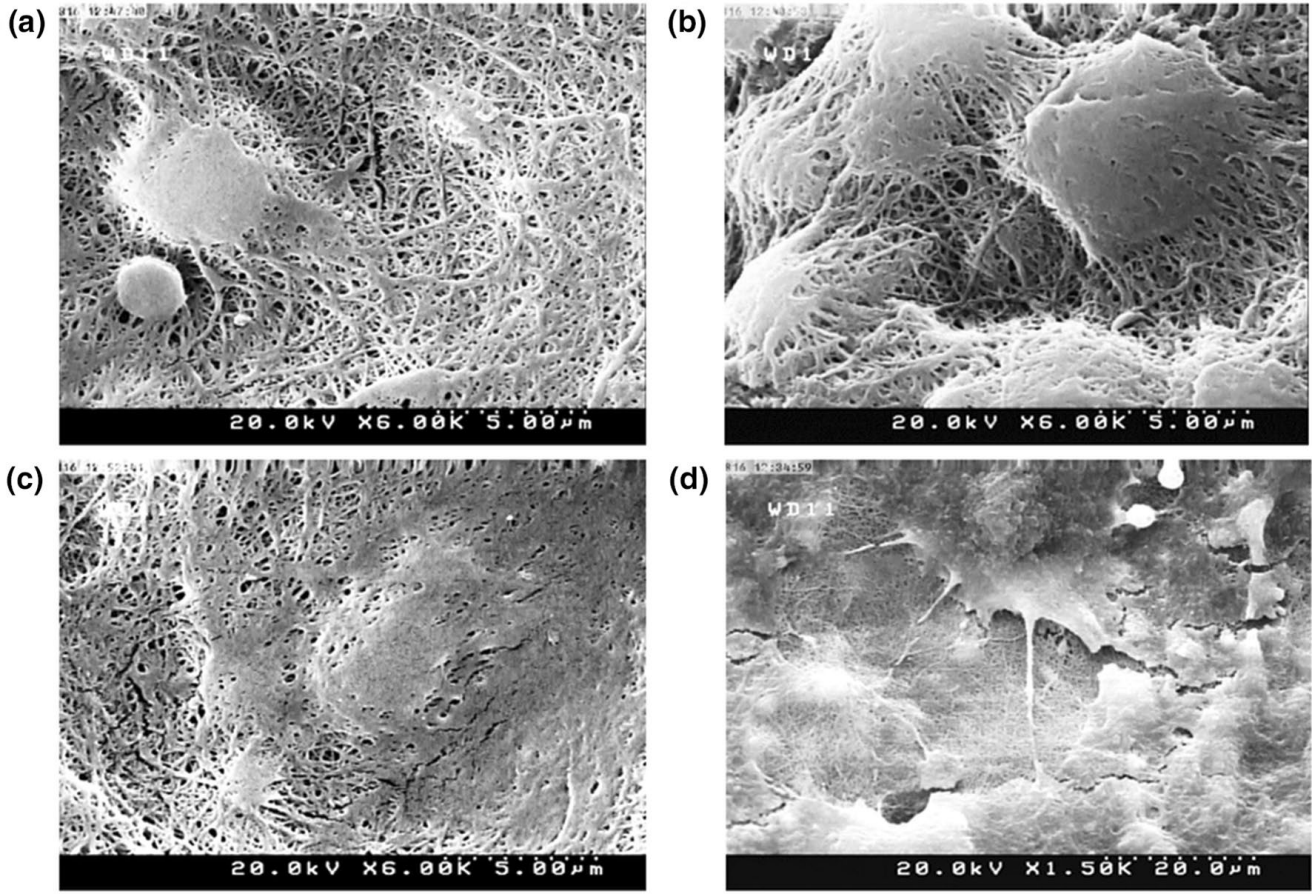
SEM images of MG63 cells cultured on PGC (**a**), PGCT_1_ (**b**), PGCT_3_ (**c**) and PGCT_5_ (**d**) samples after 72 h incubation

Cell morphology of adherent cells on the substrates after 3 days was studied by FE-SEM analysis, as shown in Fig. 6. SEM images showed more cell contacts and extent of filopodia and lamellipodia on PGCT_3_ and PGCT_5_ compared to other samples and control. The cells displayed a flat and well-spread morphology on these samples, which was an indication for their better interaction on the surface. Cell morphology could depend on surface roughness and hydrophilicity. A higher secretion of osteocalcin, TGFβ1 and prostaglandin E2 on rough surfaces was detected, which could lead to higher maturity of the cells morphologically (Nandakumar et al. 2009). According to the AFM and contact angle results, the increase of β-TCP affected the roughness and hydrophilicity of the surface of the substrates and consequently a better cell–substrate interaction was detected.

### Gene expression

**Fig. 7:**
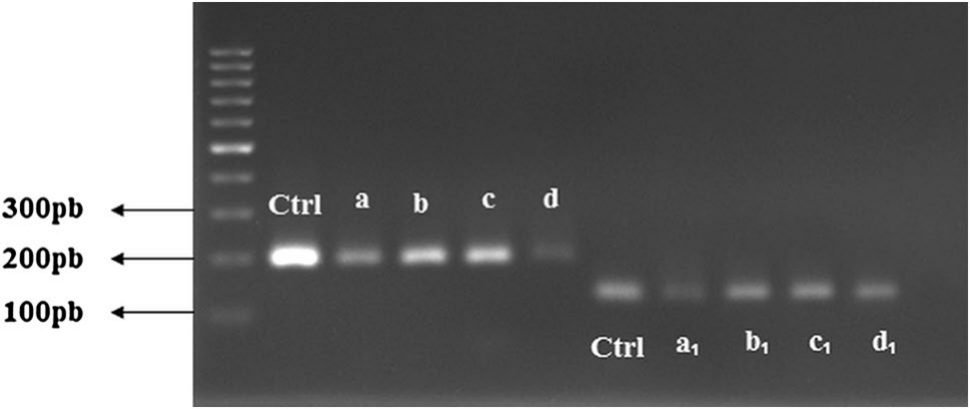
PCR amplification for collagens I (a–d) and ALP (a_1_–d_1_) after 21 days

Bone formation on osteoblasts depends on their ability to synthesize and secrete bone-specific extracellular matrix proteins such as osteocalcin and type I collagen (Aubin 1998). In the current study, the substrates were examined relating to formation of two osteogenic makers including ALP for early stage differentiation and Type I collagen for late stage differentiation of MG63 cells. Table 2 presents the primer sequences that were used for gene expression experiment. It was observed that MG63 osteoblast-like cells grown on PGCT_1_, PGCT_3_ and PGCT_5_ show notable characteristics of maturation compared to those grown on PGC as indicated by the expression of type I collagen. With addition of β-TCP up to 3%, the expression of collagen I was increased. This could be ascribed to release of Ca^2+^ from composite nanofibers. Additionally, it was observed that collagen I gene expression was the highest on those samples containing 3% β-TCP after 21 days. This may be explained by the sustained release of calcium ions from biodegradable β-TCP. In all samples, ALP expression was found to be not significantly different between the control and modified samples (Fig. 7).

**Table 2:** Oligonucleotide primers used for PCR amplification

Gene	Primer sequence: sense/antisense
Collagen type 1	5′-TCCCAGAACATCACCTACCA-3′ 5′-ATTCAATCACTGTCTTGCCC-3′
ALP (alkaline phosphatase activity)	5′-ACTCCCATCTCCTTA CCT CT-3′ 5′-TCTTGGAGTGAGTGAGTGAG-3′

### Antibacterial efficiency

**Fig. 8:**
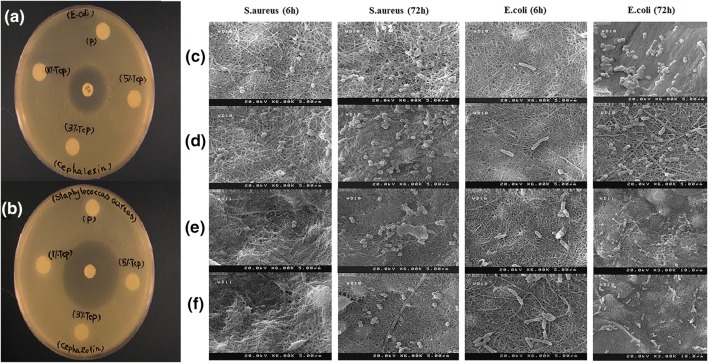
Antibacterial efficacies of the samples against *S. aureus* and *E. coli* evaluated by zone of inhibition assay (**a**, **b**). Scanning electron micrographs of *S. aureus* and *E. coli* adhered on PGC (**c**), PGCT_1_ (**d**), PGCT_3_ (**e**) and PGCT_5_ (**f**) samples after 6 and 72 h

Chitosan is known for its antibacterial properties against several organisms including *Escherichia coli* and *Staphylococcus aureus* (Bratskaya et al. 2007; Chua et al. 2008). In this study, both Gram-positive (*S. aureus*) and Gram-negative (*E. coli*) bacteria were used to evaluate the antibacterial activity on the samples by analyzing the inhibition zone. Despite the antibacterial activity of chitosan, none of the samples produced any noticeable inhibition zone against either species (*E. coli* and *S. aureus*) Figs. 8a–b. There are several factors which can influence the antibacterial activity of chitosan, including the molecular weight, degree of deacetylation and chitosan concentration (Chen et al. 2002; Mellegård et al. 2011; Chen and Zhao 2012). It can be assumed that the amount of chitosan used in the substrates was not enough to create any antibacterial activity.

Figure 8 shows the scanning electron microscopy (SEM) micrographs of the bacterial adhesion after 6 h and 72 h. In all samples, no significant difference in *S. aureus* and *E. coli* adhesion on different substrates was detected. However, bacterial adhesion was increased as the amount of β-TCP was increased after 72 h and a large population of bacteria was observed on the samples. Bacterial adhesion to biomaterial surfaces can lead to bacterial infections, which can be difficult to treat with antibiotics (Al-Ahmad et al. 2011). Generally, it is accepted that due to the enhanced contact area, as a result of increase in surface roughness, bacterial adhesion may be promoted. However, it is important to note that the size and shape of bacterial cells and other environmental factors can play a crucial role in bacterial adhesion and biofilm formation (Renner and Weibel 2011). In a similar study using PLLA/TCP scaffolds, it has been observed that addition of TCP unexpectedly could decrease bacterial adhesion (Al-Ahmad et al. 2011). Xing et al. (2015) found that bacterial adhesion on TiZr dental implant abutment is highly correlated to the surface roughness. In contrast, Lin et al. (2013) observed that increase in the roughness of ceramic surfaces could not assist biofilm formation of *S. mutans*. These conflicting results may be due to the levels of roughness, bacterial strain, culture conditions and distinctive material compositions (Song et al. 2015).

### In vitro degradation and swelling

**Fig. 9:**
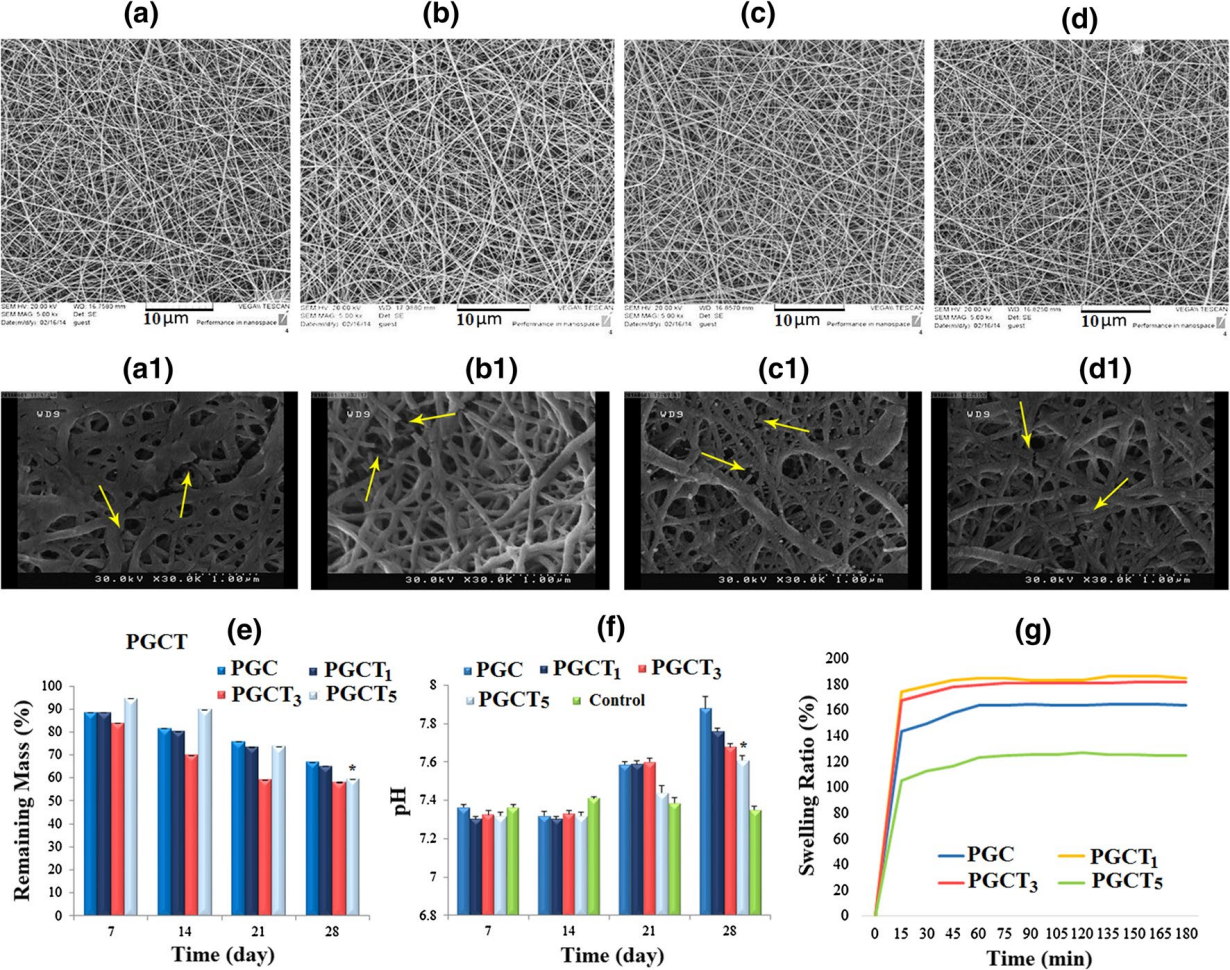
Scanning electron micrographs of membrane samples before PGC (**a**), PGCT_1_ (**b**), PGCT_3_ (**c**) and PGCT_5_ (**d**) and after PGC (**a**_**1**_), PGCT_1_ (**b**_**1**_), PGCT_3_ (**c**_**1**_) and PGCT_5_ (**d**_**1**_) degradation (4 weeks of soaking in PBS). The weight loss (**e**) and micro-environmental pH variation (**f**) of samples during 4 weeks incubation in PBS, *P < 0.05, compared to other samples. Dynamic swelling behavior of the samples after immersion in PBS at 37 °C (**g**)

For a bone substrate fabricated from biodegradable materials, it is necessary to obtain a degradation rate that allows load transfer to the healing bone. The biomaterials are usually incubated in PBS to simulate the in vivo conditions (Zhang and Ma 2001; Costa-Pinto et al. 2014). To assess the degradation trends of designed membranes, degradation tests and pH measurements were performed over a period of 4 weeks, while the membranes were incubated in 10 mL PBS at 37 °C. The morphology of the substrates before and after immersion in PBS was assessed by SEM. The samples were washed with deionized water before being analyzed by SEM. The results showed that the degradation time influenced the pore size and morphology of the substrates. After 4 weeks, most pores collapsed, bigger pores were formed and some microcracks appeared on the surfaces of the samples (Fig. 9a–d, a_1_–d_1_). The weight loss and pH variation of the PBS solution are shown in Fig. 9e–f. The results showed no significant weight loss or pH changes during the first week of the experiment. After 2 weeks of incubation, all the samples showed negligible weight loss; however, there was no significant change in pH. However, with the incorporation of β-TCP, the biodegradation rate of the membranes was accelerated. After 4 weeks, the weight loss of PGCT_5_ was remarkably higher compared to the PGC, PGCT1 and PGCT3 membrane samples. The weight losses were found to be 67%, 65%, 59% and 57% for PGC, PGCT_1_, PGCT_3_ and PGCT_5_, respectively, after 4 weeks of incubation. The fast degradation of PGCT_5_ might be due to the presence of higher β-TCP content which also affected the surface hydrophilicity of the sample. None of the samples showed significant pH changes over the period of 2 weeks’ degradation. However, the pH of all groups was increased between weeks 2 and 4. An increase in pH was observed for PGC (approximately, from 7.4 to 7.9) and PGCT_1,_ PGCT_3_ (approximately, from 7.4 to 7.7) at week 4 (Fig. 9f). The pH of the PCGT_5_ solution was almost stable during the experiment. After the 4 weeks period, the samples were left in PBS solution and completely degraded after 2 months. According to the results, the samples containing higher amount of β-TCP showed greater weight loss which is in agreement with previous reports (Heidemann et al. 2001; Debusscher et al. 2009; Huang et al. 2011). This could be correlated to the increased hydrophilicity of the substrates containing higher β-TCP, as it is known that hydrophilic substrates would have greater weight loss than hydrophobic substrates (Cao et al. 2012). β-TCP also may be degraded to Ca^2+^ and PO4^3−^ during the degradation process, which can provide mineral elements for bone regeneration. The degradation products also may counteract alteration in the pH during the degradation process. It could therefore be advantageous to regulate the degradation rate of substrates or neutralize any pH changes caused by polymer degradation through optimizing the amount of β-TCP (Heidemann et al. 2001; Lu et al. 2012).

Swelling is another consideration when fabricating a scaffold for tissue regeneration, as it influences the absorption of body fluids, the transfer of cell nutrients, and metabolites throughout the materials and can generate unnecessary stress on surrounding tissues (Jin et al. 2015). The results showed that the swelling of samples was stable after 3 h (Fig. 9g). However, β-TCP enhanced hydrophilicity and swelling of the samples, but the swelling with 5% β-TCP decreased compared to other samples. This can be related to the cross-linking effect of β-TCP (Aryaei et al. 2015). It has been found that with increase in β-TCP concentration; more cross-linked networks would form between polymeric chains which in turn reduce the substrate pore size. This probably has a negative influence on the degree of water intake by the substrates (Khan and Ranjha 2014).

## Conclusion

Calcium phosphate-based composite materials have been investigated as a superior candidate for bone regeneration in GTR/GBR. In this study, different PCL/chitosan/gelatin membrane samples by addition of various amounts of β-TCP were fabricated to investigate their biocompatibility and osteogenic properties for potential GTR/GBR applications. The results showed that MG63 cell attachment, proliferation rate, morphology, type I collagen gene expression, degradation rate and swelling as well as mechanical properties were optimized in a sample containing 3% β-TCP. It was found that the amounts of β-TCP used in this study could create no significant antibacterial effect on the composite samples. In summary, PCL/chitosan/gelatin/β-TCP substrate with 3% β-TCP would be considered a promising material candidate for generation of bone in GBR applications.

## References

[CR1] Akin FA, Zreiqat H, Jordan S, Wijesundara MB, Hanley L (2001). Preparation and analysis of macroporous TiO2 films on Ti surfaces for bone–tissue implants. J Biomed Mater Res Part A.

[CR2] Al-Ahmad A (2011). Comparison of bacterial adhesion and cellular proliferation on newly developed three-dimensional scaffolds manufactured by rapid prototyping technology. J Biomed Mater Res Part A.

[CR3] Aryaei A, Liu J, Jayatissa AH, Jayasuriya AC (2015). Cross-linked chitosan improves the mechanical properties of calcium phosphate–chitosan cement. Mater Sci Eng C.

[CR4] Aubin JE (1998). Bone stem cells. J Cell Biochem.

[CR5] Bosworth LA, Downes S (2010). Physicochemical characterisation of degrading polycaprolactone scaffolds. Polym Degrad Stab.

[CR6] Branemark P-I (1983). Osseointegration and its experimental background. J Prosthet Dent.

[CR7] Bratskaya S (2007). Adhesion and viability of two enterococcal strains on covalently grafted chitosan and chitosan/κ-carrageenan multilayers. Biomacromolecules.

[CR8] Cao L (2012). Degradation and osteogenic potential of a novel poly (lactic acid)/nano-sized β-tricalcium phosphate scaffold. Int J Nanomed.

[CR9] Cao W, Li H, Zhang J, Li D, Acheampong DO, Chen Z, Wang M (2013). Periplasmic expression optimization of VEGFR2 D3 adopting response surface methodology: antiangiogenic activity study. Protein Expr Purif.

[CR10] Chen JL, Zhao Y (2012). Effect of molecular weight, acid, and plasticizer on the physicochemical and antibacterial properties of β-chitosan based films. J Food Sci.

[CR11] Chen Y-M, Chung Y-C, Woan Wang L, Chen K-T, Li S-Y (2002). Antibacterial properties of chitosan in waterborne pathogen. J Environ Sci Health Part A.

[CR12] Chiapasco M, Casentini P, Zaniboni M (2009). Bone augmentation procedures in implant dentistry. Int J Oral Maxillofac Implant.

[CR13] Chua P-H, Neoh K-G, Kang E-T, Wang W (2008). Surface functionalization of titanium with hyaluronic acid/chitosan polyelectrolyte multilayers and RGD for promoting osteoblast functions and inhibiting bacterial adhesion. Biomaterials.

[CR14] Coïc M, Placet V, Jacquet E, Meyer C (2009). Mechanical properties of collagen membranes used in guided bone regeneration: a comparative study of three models. Revue de Stomatologie et de Chirurgie Maxillo-faciale.

[CR15] Costa-Pinto AR (2014). In vitro degradation and in vivo biocompatibility of chitosan–poly (butylene succinate) fiber mesh scaffolds. J Bioact Compat Polym.

[CR16] Dahlin C, Sennerby L, Lekholm U, Linde A, Nyman S (1989). Generation of new bone around titanium implants using a membrane technique: an experimental study in rabbits. Int J Oral Maxillofac Implant.

[CR17] Debusscher F, Aunoble S, Alsawad Y, Clement D, Le Huec J-C (2009). Anterior cervical fusion with a bio-resorbable composite cage (beta TCP–PLLA): clinical and radiological results from a prospective study on 20 patients. Eur Spine J.

[CR18] Degidi M, Scarano A, Piattelli A (2003). Regeneration of the alveolar crest using titanium micromesh with autologous bone and a resorbable membrane. J Oral Implantol.

[CR19] Dimitriou R, Mataliotakis GI, Calori GM, Giannoudis PV (2012). The role of barrier membranes for guided bone regeneration and restoration of large bone defects: current experimental and clinical evidence. BMC Med.

[CR20] Gottlow J (1993). Guided tissue regeneration using bioresorbable and non-resorbable devices: initial healing and long-term results. J Periodontol.

[CR21] Hämmerle CH, Karring T (1998). Guided bone regeneration at oral implant sites. Periodontology.

[CR22] Heidemann W (2001). Degradation of poly (D, L) lactide implants with or without addition of calcium phosphates in vivo. Biomaterials.

[CR23] Huang J, Zhang L, Chu B, Peng X, Tang S (2011). Repair of bone defect in caprine tibia using a laminated scaffold with bone marrow stromal cells loaded poly (L-lactic acid)/β-tricalcium phosphate. Artif Organs.

[CR24] Hürzeler MB, Kohal RJ, Naghshbandl J, Mota LF, Conradt J, Hutmacher D, Caffesse RG (1998). Evaluation of a new bioresorbable barrier to facilitate guided bone regeneration around exposed implant threads: an experimental study in the monkey. Int J Oral Maxillofac Surg.

[CR25] Ilizarov GA (1989). The tension-stress effect on the genesis and growth of tissues: part I. The influence of stability of fixation and soft-tissue preservation. Clin Orthop Relat Res.

[CR26] Jeong SI, Kim B-S, Kang SW, Kwon JH, Lee YM, Kim SH, Kim YH (2004). In vivo biocompatibilty and degradation behavior of elastic poly (l-lactide-co-*ε*-caprolactone) scaffolds. Biomaterials.

[CR27] Jiang T, Carbone EJ, Lo KWH, Laurencin CT (2015). Electrospinning of polymer nanofibers for tissue regeneration. Prog Polym Sci.

[CR28] Jin RM, Sultana N, Baba S, Hamdan S, Ismail AF (2015). Porous pcl/chitosan and nha/pcl/chitosan scaffolds for tissue engineering applications: fabrication and evaluation. J Nanomater.

[CR29] Kato E, Lemler J, Sakurai K, Yamada M (2014). Biodegradation property of beta-tricalcium phosphate-collagen composite in accordance with bone formation: a comparative study with Bio-Oss Collagen^®^ in a rat critical-size defect model. Clin Implant Dent Relat Res.

[CR30] Khan S, Ranjha NM (2014). Effect of degree of cross-linking on swelling and on drug release of low viscous chitosan/poly (vinyl alcohol) hydrogels. Polym Bull.

[CR31] Kim TK, Yoon JJ, Lee DS, Park TG (2006). Gas foamed open porous biodegradable polymeric microspheres. Biomaterials.

[CR32] Kinoshita Y (2008). Alveolar bone regeneration using absorbable poly (L-lactide-co-*ɛ*-caprolactone)/β-tricalcium phosphate membrane and gelatin sponge incorporating basic fibroblast growth factor. Int J Oral Maxillofac Surg.

[CR33] Ko H-F, Sfeir C, Kumta PN (2010). Novel synthesis strategies for natural polymer and composite biomaterials as potential scaffolds for tissue engineering philosophical transactions of the Royal Society of London A: mathematical. Phys Eng Sci.

[CR34] Kong M, Chen XG, Xing K, Park HJ (2010). Antimicrobial properties of chitosan and mode of action: a state of the art review. Int J Food Microbiol.

[CR35] Lam CX, Teoh SH, Hutmacher DW (2007). Comparison of the degradation of polycaprolactone and polycaprolactone–(β-tricalcium phosphate) scaffolds in alkaline medium. Polym Int.

[CR36] Li W-J, Cooper JA, Mauck RL, Tuan RS (2006). Fabrication and characterization of six electrospun poly (*α*-hydroxy ester)-based fibrous scaffolds for tissue engineering applications. Acta Biomater.

[CR37] Lin HY, Liu Y, Wismeijer D, Crielaard W, Deng DM (2013). Effects of oral implant surface roughness on bacterial biofilm formation and treatment efficacy. Int J Oral Maxillofac Implant.

[CR38] Lindfors LT, Tervonen EA, Sándor GK, Ylikontiola LP (2010). Guided bone regeneration using a titanium-reinforced ePTFE membrane and particulate autogenous bone: the effect of smoking and membrane exposure. Oral Surg Oral Med Oral Pathol Oral Radiol Endodontol.

[CR39] Lu L (2012). Biocompatibility and biodegradation studies of PCL/β-TCP bone tissue scaffold fabricated by structural porogen method. J Mater Sci Mater Med.

[CR40] Maji K, Dasgupta S (2017). Effect of βtricalcium phosphate nanoparticles additions on the properties of gelatin–chitosan scaffolds. Bioceram Dev Appl.

[CR41] Mellegård H, Strand S, Christensen B, Granum P, Hardy S (2011). Antibacterial activity of chemically defined chitosans: influence of molecular weight, degree of acetylation and test organism. Int J Food Microbiol.

[CR42] Mota J (2012). Chitosan/bioactive glass nanoparticle composite membranes for periodontal regeneration. Acta Biomater.

[CR43] Nandakumar A, Yang L, Habibovic P, van Blitterswijk C (2009). Calcium phosphate coated electrospun fiber matrices as scaffolds for bone tissue engineering. Langmuir.

[CR44] Pineda LM, Büsing M, Meinig RP, Gogolewski S (1996). Bone regeneration with resorbable polymeric membranes. III. Effect of poly (L-lactide) membrane pore size on the bone healing process in large defects. J Biomed Mater Res Part A.

[CR45] Postlethwaite AE, Seyer JM, Kang AH (1978). Chemotactic attraction of human fibroblasts to type I, II, and III collagens and collagen-derived peptides. Proc Natl Acad Sci.

[CR46] Reddi AH, Wientroub S, Muthukumaran N (1987). Biologic principles of bone induction. Orthop Clin N Am.

[CR47] Ren K, Wang Y, Sun T, Yue W, Zhang H (2017). Electrospun PCL/gelatin composite nanofiber structures for effective guided bone regeneration membranes. Mater Sci Eng C.

[CR48] Renner LD, Weibel DB (2011). Physicochemical regulation of biofilm formation. MRS Bull.

[CR49] Sarasam A, Madihally SV (2005). Characterization of chitosan–polycaprolactone blends for tissue engineering applications. Biomaterials.

[CR50] Schmidmaier G, Baehr K, Mohr S, Kretschmar M, Beck S, Wildemann B (2006). Biodegradable polylactide membranes for bone defect coverage: biocompatibility testing, radiological and histological evaluation in a sheep model. Clin Oral Implant Res.

[CR51] Sheikh Z, Najeeb S, Khurshid Z, Verma V, Rashid H, Glogauer M (2015). Biodegradable materials for bone repair and tissue engineering applications. Materials.

[CR52] Shim J-H (2012). Fabrication of blended polycaprolactone/poly (lactic-co-glycolic acid)/β-tricalcium phosphate thin membrane using solid freeform fabrication technology for guided bone regeneration. Tissue Eng Part A.

[CR53] Smith I, Liu X, Smith L, Ma P (2009). Nanostructured polymer scaffolds for tissue engineering and regenerative medicine. Wiley Interdiscip Rev Nanomed Nanobiotechnol.

[CR54] Song F, Koo H, Ren D (2015). Effects of material properties on bacterial adhesion and biofilm formation. J Dent Res.

[CR55] Takahashi Y, Tabata Y (2004). Effect of the fiber diameter and porosity of non-woven PET fabrics on the osteogenic differentiation of mesenchymal stem cells. J Biomater Sci Polym Ed.

[CR56] Tsuru K, Munar M, Ishikawa K, Othman R (2013). Mechanical behavior and cell response of PCL coated α-TCP foam for cancellous-type bone replacement. Ceram Int.

[CR57] Urban IA, Jovanovic SA, Lozada JL (2009). Vertical ridge augmentation using guided bone regeneration (GBR) in three clinical scenarios prior to implant placement: a retrospective study of 35 patients 12 to 72 months after loading. Int J Oral Maxillofac Implant.

[CR58] Xing R, Lyngstadaas SP, Ellingsen JE, Taxt-Lamolle S, Haugen HJ (2015). The influence of surface nanoroughness, texture and chemistry of TiZr implant abutment on oral biofilm accumulation. Clin Oral Implant Res.

[CR59] Xu C, Lei C, Meng L, Wang C, Song Y (2012). Chitosan as a barrier membrane material in periodontal tissue regeneration. J Biomed Mater Res Part B Appl Biomater.

[CR60] Zhang R, Ma P (2001) Composite scaffolds for bone tissue engineering: degradation. In: 47th Annual meeting, Orthopaedic Research Society, San Francisco

[CR61] Zhou H, Lee J (2011). Nanoscale hydroxyapatite particles for bone tissue engineering. Acta Biomater.

